# Regulation and Function of Syk Tyrosine Kinase in Mast Cell Signaling and Beyond

**DOI:** 10.1155/2011/507291

**Published:** 2011-05-12

**Authors:** Rodrigo Orlandini de Castro

**Affiliations:** ^1^Receptors and Signal Transduction Section, Oral Infection and Immunity Branch, National Institute of Dental and Craniofacial Research, National Institutes of Health, Bethesda, MD 20892, USA; ^2^Department of Cell and Molecular Biology and Pathogenic Bioagents, Faculade de Medicina de Ribeirão Preto, University of São Paulo, Ribeirão Preto, SP 14049-900, Brazil

## Abstract

The protein tyrosine kinase Syk plays a critical role in Fc*ε*RI signaling in mast cells. Binding of Syk to phosphorylated immunoreceptor tyrosine-based activation motifs (p-ITAM) of the receptor subunits results in conformational changes and tyrosine phosphorylation at multiple sites that leads to activation of Syk. The phosphorylated tyrosines throughout the molecule play an important role in the regulation of Syk-mediated signaling. Reconstitution of receptor-mediated signaling in Syk^−/−^ cells by wild-type Syk or mutants which have substitution of these tyrosines with phenylalanine together with *in vitro* assays has been useful strategies to understand the regulation and function of Syk.

## 1. Introduction

The clustering of the high-affinity receptor for IgE (Fc*ε*RI) initiates a series of biochemical events in mast cells or basophils that culminate with the release of preformed mediators from granules together with the synthesis and release of lipid mediators and cytokines [1–6]. The Fc*ε*RI is a member of a larger family of multisubunit immune recognition receptors (MIRRs), which also includes the T-cell receptor (TCR), the B-cell receptor (BCR), and some other Fc receptors (FcR) like Fc*γ*RI and Fc*γ*RIII [7]. These receptors have no intrinsic catalytic activity. Instead, their activation results in phosphorylation of the two tyrosines in the ITAM (immunoreceptor tyrosine-based activation motif) within the cytoplasmic tails of subunits of the receptor. ITAM-based signaling shares a common signal transduction mechanism, where the recruitment and activation of Syk or ZAP-70 is a critical step for these immunoreceptors to activate these cells [8, 9]. This paper summarizes important studies and new findings on regulation and function of Syk in mast cell signaling and some other cells.

## 2. Syk-Mediated Fc*ε*RI Activation

Fc*ε*RI is a tetrameric structure formed by the complex *αβγ*_2_ chains. The *α*-subunit binds the Fc portion of IgE at a ratio of 1 : 1 while the *β*-subunit and the disulphide-linked *γ*-subunits contain ITAMs in their cytoplasmic tail [5, 10]. Aggregation of Fc*ε*RI results in phosphorylation of the tyrosines within the ITAMs by Lyn, or another member of the Src family of tyrosine kinases that associate with the receptor. Phosphorylated ITAM then serves as a docking site for Syk; this binding of Syk through its SH2 domains results in a conformational change of Syk which allows *trans*- and autophosphorylation of its catalytic domain, as well as phosphorylation by Lyn, thereby increasing Syk enzymatic activity [2, 11].

Tyrosine-phosphorylated Syk leads to downstream propagation of signaling with phosphorylation of linker for activation of T cells (LAT), Vav, BTK, phospholipase C*γ* (PLC*γ*), and SH2 domain-containing leukocyte protein of 76 kDa (SLP-76) [12, 13]. Phosphorylated LAT functions as a signal platform providing binding sites directly for growth factor receptor-bound protein 2 (Grb2), Grb2-related adaptor downstream of Shc (Gads), and PLC*γ*, while indirectly binding for SLP-76, NCK, and other adaptors. PLC*γ* hydrolyzes phosphatidylinositol 4,5-bisphosphate (PIP_2_) in the plasma membrane to produce diacylglycerol, which activates protein kinase C (PKC), and inositol 1,4,5-triphosphate (IP_3_) [6, 14]. The binding of IP_3_ to its receptor in the endoplasmic reticulum (ER) membrane rapidly induces the first stage of calcium (Ca^2+^) mobilization, which is the transient release of Ca^2+^ from ER stores. The depletion of calcium in the ER induces formation of multimers of Stim1in the ER membrane, which move and interact directly with Orai1, a pore subunit of the Ca^2+^ channel, in the plasma membrane. Together with calcium released from ER, this induces prolonged Ca^2+^ influx through store-operated calcium release-activated calcium (CRAC) channels in the plasma membrane [15, 16]. Syk-mediated tyrosine phosphorylation of BTK, SLP-76, LAT, and PLC*γ* is essential to generate signals for sustained Ca^2+^ influx. All these events culminate with mast cell degranulation, phosphorylation of MAP kinases, and activation of nuclear factor for T-cell activation (NFAT) and nuclear factor *κ*B (NF*κ*B), which turn on cytokine synthesis.

## 3. The Syk Tyrosine Kinase

**Figure 1:**
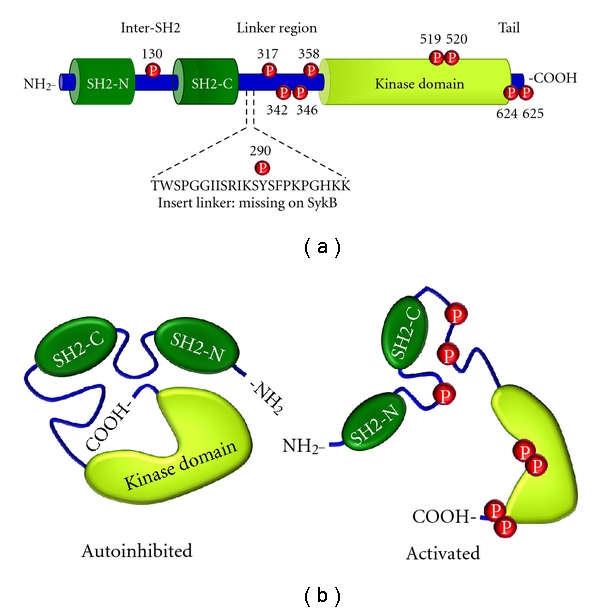
Structure of protein tyrosine kinase Syk: (a) a schematic diagram of the linear structure of Syk with the tyrosines marked that are phosphorylated after its *in vitro* activation. In the linker region there are 23 amino acids which are missing in SykB. (b) Suggested globular structure of autoinhibited (left) and activated (right) Syk with some phosphorylated tyrosines indicated.

*Syk* gene was first cloned on the basis of partial sequenced information of the 40 kDa kinase from a porcine spleen. The product of this gene was described as a member of a new nonreceptor-type protein tyrosine kinase of 72 kDa (p72*^syk^*). Interestingly, unlike *src *family protein kinases, p72*^syk^* has a second SH2 instead of an SH3 domain [17]. Molecular cloning then described a gene that encoded a 70 kDa protein tyrosine kinase associated with the *ζ* chain of the T-cell receptor, termed ZAP-70 [18]. This also had two SH2 domains and was very similar to p72*^syk^*. Those findings indicated that Syk and ZAP-70 belonged to a distinct subfamily of nonreceptor protein tyrosine kinases [19], named Syk family of protein kinases. Syk is expressed in mast cells, B cells, immature T cells, and platelets, whereas ZAP-70 is in T cells and natural killer cells [18, 20]. Structurally, both Syk and ZAP-70 have two Src-homology 2 domains (SH2) and a kinase domain. The interdomain A is located between the NH_2_-terminal SH2 (SH2-N) and COOH-terminal SH2 (SH2-C), while interdomain B or linker region is between the SH2-C and the kinase domains; following the kinase domain there is a short COOH-terminal extension (tail) [21] (Figure 1).

*In vivo*, Syk has been reported to be essential in maintaining vascular integrity and wound healing during embryogenesis. Syk^−/−^ mice showed petechiae, suffered severe hemorrhaging as embryos, and died perinatally [22, 23]. Also, irradiated mice reconstituted with Syk-deficient fetal liver showed a block in B-cell development and maturation [23]. 

In cells, Syk plays an essential role in IgE-mediated activation of mast cells. Syk^−/−^ mast cells are unable to induce Ca^2+^ mobilization, degranulate, phosphorylate MAP kinases, and activate NFAT or NF*κ*B; but transfection of these cells with Syk reconstitutes their activation [24–26]. Similarly, BCR crosslinking in Syk^−/−^ B cells also fails to induce cell activation [27]. Recent studies have shown that bone marrow-derived mast cell (BMMC) treated with siRNA targeted to Syk efficiently decreases receptor-induced cell activation [2, 28]. 

Due to structural similarities Syk and ZAP-70 have also some functional redundancies. For example, expression of either ZAP-70 or Syk reconstitutes receptor-induced cell activation in Syk-negative mast cells and B cells [26, 27, 29]; however, ZAP-70 but not Syk requires CD45 to efficiently induce Fc*ε*RI-induced signaling responses in Syk-negative mast cells [29]. Additionally, in CD45-deficient T cells, Syk but not ZAP-70 restores the TCR signaling pathway [30]. CD45 is a critical component of TCR, acting as a positive regulator of Src family protein tyrosine kinases, such as Lck. Activation of TCR leads to dephosphorylation of negative sites of Lck which then activates ZAP-70. These studies demonstrate that binding of Syk to the phosphorylated ITAM is enough to trigger its enzymatic activity while ZAP-70 requires an additional stimulatory input from other tyrosine kinases.

## 4. Phosphorylated ITAM-Induced Syk Activation

**Figure 2:**
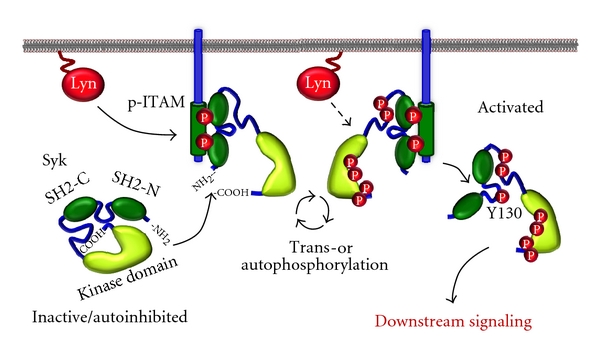
ITAM-based Syk activation: activation of the immunoreceptor, here represented by a *γ*-subunit of Fc*ε*RI, results in the phosphorylation of the ITAMs by Lyn or other Src kinase that associates with the receptor. This recruits the cytoplasmic Syk which binds to the phosphorylated ITAM (p-ITAM) resulting in conformational changes that disrupt its autoinhibited structure, which increase the kinase activity of Syk. After activation, Syk is tyrosine phosphorylated essentially by trans- or autophosphorylation with some participation of other tyrosine kinases. The phosphorylation of Tyr-130 releases the kinase from the receptor allowing Syk to phosphorylate its targets in the cytoplasm of the cell.

ITAM-based signaling has been extensively characterized and serves as a model for receptor-mediated signal transduction [8, 9]. The ITAM consensus sequence is D/E-(X)_2_-Y-(X)_2_-L/I-(X)_6/7_-Y-(X)_2_-L/I, where the two tyrosines residues are phosphorylated upon receptor activation. The *β*- and *γ*-subunits of Fc*ε*RI complex contain ITAM on their cytoplasmic tails. Phosphorylated ITAM (p-ITAM) serves as the docking site for the two SH2 domains of Syk [31–34]. The association of Syk with p-ITAM occurs with an antiparallel binding orientation: the SH2-N of Syk binds to the COOH-terminal phosphotyrosine of the ITAM while the SH2-C binds to the NH_2_-terminal phosphotyrosine [31, 32] (Figure 2). *In vitro* experiments using synthetic peptides corresponding to the *β*- or *γ*-ITAM of Fc*ε*RI showed that Syk binds preferentially to the biphosphorylated *γ*-ITAM [32]. The SH2 domain-mediated binding of Syk/ZAP-70 to phosphorylated ITAM in subunits of immune receptors is critical for activation of these cells; the expression in cells of truncated Syk containing just the two SH2 domains has a dominant-negative effect that inhibits binding of endogenous Syk and decreases receptor signaling [35]. Although both SH2 domains of Syk are required for its binding to p-ITAM, the SH2-C binds stronger to the phosphorylated *γ*-ITAM than the SH2-N. The binding of Syk to the p-ITAM results in conformational changes of Syk exposing its COOH-terminal region [36] by disrupting the COOH-terminal/SH2-interdomain interactions [37, 38]. Interaction of the COOH-terminal with SH2 interdomain stabilizes the autoinhibitory structure of the enzyme; the binding to p-ITAM induces a conformational change that “opens” the molecule resulting in Syk activation [25]. In addition, Syk itself can phosphorylate the ITAMs creating a positive feedback loop during the initial steps of receptor-mediated Syk activation [39] (Figure 2).

## 5. Autoinhibition of Syk/Zap-70

Structural studies of ZAP-70 suggest an autoinhibitory state for Syk family kinases [37, 38]. Several tyrosine residues throughout the molecule can regulate Syk/ZAP-70 enzymatic activity; phosphorylation of these can stabilize the active form, whereas in the nonphosphorylated state the same residues may contribute to autoinhibition of the enzyme. In the inactive ZAP-70, the hydrogen-bonding network is stabilized by a hydrophobic cluster formed by Pro-396, Tyr-397, and Tyr-474 in the kinase domain and Tyr-319 in the linker region, whereas, in the active form, the hydrogen-bonding network and hydrophobic interactions are disrupted [37]. This structural study of ZAP-70 was with a protein that had the COOH-terminal region missing, and the crystal structure of only the SH2 domains of Syk has been determined. However, it is predicted that the two tyrosines in the tail of ZAP-70 interact with the *α*-helix of the C-lobe of the kinase domain and the inter-SH2 domain regions; these interactions help to stabilize the autoinhibitory form of Syk/ZAP-70 [25, 38].

## 6. Regulation of Syk Function by Its Tyrosine Phosphorylation

Activation of Syk leads to its tyrosine phosphorylation due mostly to autophosphorylation with some contribution by other tyrosine kinases [40]. Analysis of activated Syk has indentified ten tyrosines residues which are autophosphorylated *in vitro* [41] (Figure 1); several of these are also found phosphorylated in mast cells after receptor activation [42]. These phosphotyrosines can regulate Syk function and also generate potential docking sites for other molecules. 

One of these phosphotyrosines at Tyr-130 is located in the inter-SH2 domain and is an early site of Syk autophosphorylation. Phosphorylation of Tyr-130 regulates the binding of Syk to the p-ITAM by dissociating Syk from the receptor. Substitution of Tyr-130 with Phe enhances binding of Syk to the receptor while this is greatly reduced by substitution with Glu [43, 44]. Studies also suggest that phosphorylation of Tyr-130 destabilizes inter-SH2 domain structure, which alters the orientation and increases the distance between the two SH2 domains beyond the limit permitted for their functional binding to p-ITAM [44].

The linker region plays a critical role in regulation of Syk function. An alternative spliced form of Syk, named SykB, lacks 23 amino acids, known as “linker insert,” in the linker region (Figure 1). This linker insert enhances the binding of Syk to the p-ITAM, which helps to explain why SykB is inefficient in reconstituting Fc*ε*RI signaling in Syk-deficient mast cells [45]. Within the linker insert is Tyr-290, which is autophosphorylated *in vitro* [41]. Substitution of Tyr-290 of Syk with Phe does not affect Syk-mediated downstream signaling of either Fc*ε*RI or TCR [45]. Also, BCR-mediated NFAT activation in Syk-deficient DT40 cells expressing Syk-Y290F was similar to cells with wild-type Syk [46]. Therefore, Tyr-290 has no obvious effect on Syk-mediated signaling. 

Three conserved tyrosines, Tyr-317, Tyr-342, and Tyr-346, are located in the linker region. These are found phosphorylated in both Syk and ZAP-70 after receptor activation [42, 47]. It has been reported that Tyr-317 is a negative regulatory site of Syk function [48]. Substitution of Tyr-317 with Phe increases Syk kinase activity and its autophosphorylation capacity. *In vivo*, expression of Syk-Y317F enhances mast cell degranulation and phosphorylation of PLC*γ*1 and PLC*γ*2 without affecting its binding to the p-ITAM [48]. Also, expression of Syk-Y317F in DT40 B cells enhances BCR-mediated activation of both transcription factors NFAT and Elk-1 [49]. Phosphorylation of Tyr-317 not only negatively regulates Syk function but also creates a binding site for protein ubiquitin ligases of Cbl family, which target receptors and tyrosine kinases [50]. Among the three members of Cbl family proteins two of them, c-Cbl and Cbl-b, are expressed in hematopoietic cells [51]. The absence of Cbl-b enhances mast cell degranulation, Ca^2+^ influx, and synthesis of cytokines such as TNF-*α* and IL-6 [52, 53]. These are mostly due to increased phosphorylation of both Syk and receptor phosphorylation in Cbl-b^−/−^ mast cells. Studies using c-Cbl^−/−^ mast cells show that c-Cbl has more effect on phosphorylation of MAP kinases but does not affect Syk phosphorylation [53]. Therefore phosphorylation of Tyr-317 negatively regulates Syk function, and this also creates a binding site for Cbl-b which leads to ubiquitination and degradation of Syk. 

Tyrosines 342 and 346 are phosphorylated after receptor activation in mast cells [54]. These tyrosines have been reported to be involved in the interaction of Syk with PLC*γ* and Vav. Thus, in COS cells expression of Syk with both Tyr-342 and Tyr-346 mutated to Phe results in loss of interaction of Syk with PLC*γ* [55]. Experiments using two-hybrid system suggest that phosphorylated Tyr-342 of Syk is a binding site for VAV [56]. Functional analysis of each of these tyrosines shows that substitution of Tyr-346 with Phe impairs Syk-mediated phosphorylation of SLP-76, LAT, and PLC-*γ*2, calcium mobilization, and mast cell degranulation [54]. This can be as a result of decreased kinase activity and reduced binding of mutated Syk to phosphorylated *γ*-ITAM. Interestingly, Tyr-346 substitution with Phe has minimal effects on receptor-mediated mast cell degranulation. Another tyrosine phosphorylated in the linker region is Tyr-358. This is autophosphorylated *in vitro* [41]; but there have been no reports on the role of Tyr-358 in Syk function.

Aggregation of Fc*ε*RI results in phosphorylation of two adjacent tyrosines in the activation loop of Syk kinase domain, Tyr-519/Tyr-520. Both of these are essential for Syk-mediated receptor signaling in mast cells [57]. Tyr-519 has more impact on histamine release, while Tyr-520 is more involved in phosphorylation of PLC*γ*2. However, *in vitro *experiments show that substitution of one or both of these activation loop tyrosines with Phe does not affect the kinase activity of Syk nor its binding to the p-ITAM [57]. Other studies demonstrate that the binding of mAb AA4, an antibody that recognizes a mast cell-specific ganglioside on the cell surface [58], results in the strong tyrosine phosphorylation of cellular proteins including Syk, but not histamine release or phosphorylation of the activation loop tyrosines [40]. It is suggested that phosphorylation of the activation loop tyrosines occurs mostly by auto-transphosphorylation with some participation of other tyrosine kinases in the initiation of the phosphorylation process [40, 59]. Therefore, the phosphorylation of activation loop tyrosines is critical for Syk-mediated Fc*ε*RI signaling in mast cells. Unlike Syk, mutation of Tyr-493, analogous to Tyr-520 of Syk, abrogates the *in vitro *kinase activity of ZAP-70 [60]. This disparity in the *in vitro* function of the activation loop tyrosines of Syk and ZAP-70 can be explained by differences in the enzymatic activity of both proteins. For example, the kinase activity of ZAP-70 but not Syk depends on the presence of Lck for its initial activation, and the enzymatic activity of Syk is at least 100-fold greater than that of ZAP-70 [57, 60, 61]. 

The COOH-terminal region of Syk has three conserved tyrosines (Tyr-623, Tyr-624, and Tyr-625); the last two of which are also conserved in human ZAP-70 [25]. Tyr-624 and Tyr-625 are phosphorylated in both Syk and ZAP-70 by *in vitro *autophosphorylation or following receptor stimulation [41, 42, 62, 63]. Mutation of these three Tyr to Phe disrupts the autoinhibitory state of Syk which allows it to be more tyrosine phosphorylated by another tyrosine kinase in cells, which activates Syk and results in autophosphorylation [25]. Without such phosphorylation by another tyrosine kinase, these mutations result in Syk that has very low enzymatic activity and capacity for autophosphorylation. Therefore, mutation of these tyrosines results in Syk which functionally is more similar to ZAP-70 in its requirement for another tyrosine kinase for activation. In mast cells this mutated Syk is less efficient in Fc*ε*RI signaling because of decreased binding to phosphorylated ITAM independent of phosphorylation of Tyr-130. Together with the decreased kinase activity, this leads to reduced mast cell degranulation, decreased phosphorylation of MAP kinases, and lower activation of NFAT and NF*κ*B. Other results strongly suggest that phosphorylation of COOH-terminal tyrosines of Syk creates a docking site for SLP-65 in B cells, although it is not clear if this is a result of direct binding or if other adaptors could mediate this interaction [63].

## 7. Direct and Indirect Negative Regulation of Syk Signaling

The regulation of Syk function also includes the downregulation of its activity after receptor activation; this process involves the participation of protein phosphatases and ubiquitin ligases. Cbl-b-mediated Syk ubiquitination and degradation has been the most characterized mechanism to downregulate Syk function in receptor signaling (previously discussed). The phosphatases SHIP1, SHP1, and SHP2 are tyrosine phosphorylated after Fc*ε*RI aggregation, and this phosphorylation is independent of Syk. These phosphatases are found associated with Fc*ε*RI in mast cells [64, 65]. *In vitro* experiments show that both SHP1 and SHP2 dephosphorylate *β*- and *γ*-subunits of the Fc*ε*RI [65]. These phosphatases indirectly inhibit Syk function by limiting the intensity and duration of receptor phosphorylation which, as discussed above, leads to Syk activation. Recently, a novel histidine tyrosine phosphatase, T-cell ubiquitin ligand-2 (TULA-2), has been described to associate with Syk [66]. TULA-2, also known as ubiquitin-associated domain and SH3 domain-containing protein B (UBASH3B) or suppressor of T-cell receptor signaling-1 (Sts-1), belongs to the TULA-family of proteins [67, 68]. It has been shown that TULA-2 targets phosphotyrosines 317, 346, and 519/520 of Syk [69]. Additionally, Syk has increased phosphorylation in GPVI-stimulated platelets from TULA-deficient mice or by inhibiting TULA-2 activity in 293T cells [66, 69, 70]. T cells from TULA^−/−^ mice are hyperresponsive to TCR stimulation as a result of increased activation of ZAP-70 [71]. These findings strongly suggest that TULA-2 is a phosphatase which directly downregulates the activity of Syk family proteins.

## 8. Syk-Mediated Actin Redistribution in Mast Cells

**Figure 3:**
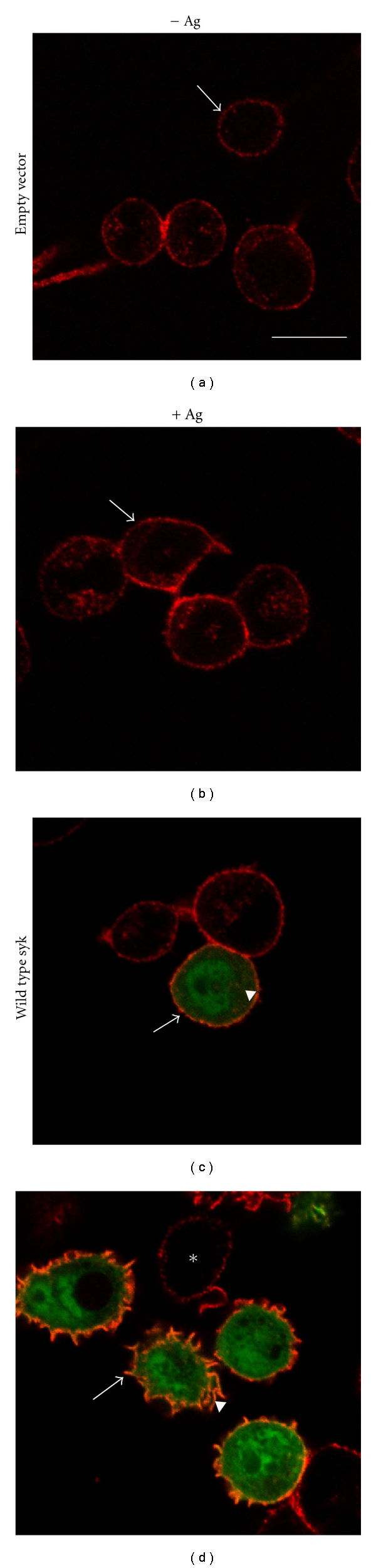
Reconstitution of Syk-mediated actin redistribution in Syk−/− mast cells: C4A2 Syk−/− mast cells were transfected with empty plasmids or plasmids that express wild-type Syk [25]. These cells were cultured with antigen-specific IgE, washed, and then either incubated with medium alone ((a) and (c)) or medium containing antigen. After 10 min, the cells were immunostained as described previously [76] using mAb anti-Syk (26B61A6) and F(ab^'^)_2_ donkey antimouse IgG conjugated with Alexa 488; actin staining was with phalloidin conjugated with Alexa-633. In nonactivated Syk−/− cells transfected with empty vector (a) or wild-type Syk (c), actin was uniformly distributed on the cell periphery (arrow). After receptor activation, in these cells expressing wild-type Syk ((d), arrow), there was redistribution of actin which follows the ruffled cell membrane, characteristic of activated mast cells; while minimal or no redistribution of actin was observed in cells that did not express Syk ((d), asterisk) or in cells transfected with empty vector (b). In nonstimulated cells expressing wild-type Syk, the Syk was found throughout the cytoplasm ((c), arrow head); after receptor activation, part of the cytoplasmic Syk was recruited to the plasma membrane and colocalized with actin ((d), arrow head). Bar: 10 *μ*m.

The RBL-2H3, a mast cell line, has been widely used as a model to study Fc*ε*RI-mediated signaling. Morphological studies show that resting RBL-2H3 cells are normally spindle shaped with small microvilli on their surface; these cells become flattened and spread and their surfaces are ruffled and plicated after receptor activation [58]. These morphological changes involve redistribution of actin and are in parallel with mediator release. Some other experiments demonstrate that these morphological changes are downstream of Syk and are Ca^2+^ dependent [2, 72]. For example, activation of Fc*ε*RI induces minimal or no morphological change in RBL-2H3 Syk^−/−^ variant mast cell line; but this is reconstituted by stable expression of Syk in these cells. Additionally, receptor-mediated redistribution of actin is observed only in cells expressing Syk after transient transfection of Syk^−/−^ cells (Figure 3). Therefore, the cellular mechanisms which result in morphological changes in mast cells are dependent on Syk activation. 

## 9. New Studies on Syk Function

Receptor-induced Syk tyrosine phosphorylation has been well characterized in hematopoietic cells [2, 12, 13, 38, 73]. However, there is little information on Syk being phosphorylated on Ser/Thr residues. Recently, an interesting study using phosphopeptide mapping and mass spectrometric analyses identified a new phosphorylated residue in the linker region of Syk, Ser-291 [46]. This is phosphorylated by PKC after BCR activation or PMA treatment in the Syk-deficient DT40 cells expressing Syk-EGFP. Substitution of Ser-291 with Ala decreases Syk-mediated BCR signaling to activate NFAT and Elk-1 but does not affect Syk kinase activity [46]. In ZAP-70, Ser-520 was predominantly phosphorylated in normal T lymphocytes and Jurkat T cells. Substitution of Ser-520 with Ala reduced the capacity of ZAP-70 to autophosphorylate and to mediate TCR-induced IL-2 promoter activation [74]. Recently, it was demonstrated that Ser-197 in the cytoplasmic tail of Ig-*α* is phosphorylated upon BCR activation which inhibits signaling [75]. Surprisingly, this study suggests that Syk is the kinase responsible for phosphorylating Ser-197 of Ig-*α*, indicating a dual-specificity kinase activity for Syk.

## 10. Conclusion

Studies have shown that Syk is a key player in mast cells and other hematopoietic cells. Several studies have also suggested the mechanisms that regulate Syk function based on its structure and phosphorylated tyrosines. Mutating Syk has been the strategy most frequently used to understand its function but, surprisingly, this could affect the autoinhibitory structure of the molecule resulting in secondary effects. For example, mutations of the three conserved tyrosines in the COOH-terminal region of Syk decreases its binding capacity to the p-ITAM due to decreased autoinhibitory structure of mutated Syk and not because of the mutation itself [25]. Also, Syk carrying either a green fluorescence protein (GFP) domain or an affinity tag at the NH_2_- or COOH-terminal seems to be more active than wild-type Syk [38, 63]. This could result in some nonspecific activities of the molecule when expressed in cells. Understanding Syk function and its interactions is important for developing pharmacological compounds that regulate its function.
